# Prognosticating post-bariatric surgery outcomes and management of postoperative recurrent weight gain and diabetes recurrence

**DOI:** 10.3389/fnut.2024.1510403

**Published:** 2024-12-23

**Authors:** He Xiao, Yudie Du, Yuanyuan Tan, Yixing Ren

**Affiliations:** ^1^Department of Gastroenterology, Affiliated Hospital of North Sichuan Medical College, Nanchong, China; ^2^Institute of Hepatobiliary Pancreatic Intestinal Diseases, North Sichuan Medical College, Nanchong, China; ^3^Department of General Surgery, Chengdu XinHua Hospital Affiliated to North Sichuan Medical College, Chengdu, China

**Keywords:** bariatric surgery, type 2 diabetes mellitus, recurrent weight gain, glucagon-like peptide-1, conversional surgery

## Abstract

Bariatric surgery stands as the most potent treatment for achieving substantial weight reduction and alleviating the complications associated with obesity. However, it is not the treatment of choice for patients with obesity combined with type 2 diabetes mellitus, and the benefit of bariatric surgery varies widely among individuals. There is a noticeable inconsistency in the outcomes following these procedures. The ability to predict how an individual will respond to bariatric surgery is a valuable asset in clinical practice. And the importance of postoperative interventions should not be underestimated. Proactive measures targeting both pre- and post-operative eating habits and lifestyle adjustments are of greater significance than the investigation into pre-operative factors alone. The judicious application of medication, endoscopic intervention and conversional surgeries after bariatric surgery can yield superior outcomes in managing recurrent weight regain and the recurrence of diabetes, albeit with consideration for the associated complication rates.

## 1 Introduction

Obesity represents a complex and widespread chronic condition, ranking among the most prevalent health issues globally (1). Bariatric surgery (BS) has become the most effective intervention for the treatment of obesity. However, many patients yet suffer from the postoperative complication of recurrent weight gain (RWG). It is important for those patients to increase their moderate to vigorous physical activity (MVPA) to mitigate RWG (2). And dietary interventions are significant since irregular eating habits are linked to RWG (3). Anti-obesity drugs can act as a potent supplementary treatment following BS, aiding in enhancing weight reduction after the procedure or averting the RWG (4, 5). Furthermore, laparoscopy intervention is a minimally invasive and safe treatment for RWG which provides sustainable weight loss (6). Conversion of previous BS may be necessary in instances of RWG and the presence of complications while conversional surgery has greater morbidity than primary surgery (7).

Furthermore, bariatric surgery is also capable of alleviate type 2 diabetes mellitus (T2DM). A range of predictive models have been utilized to forecast the remission of T2DM, including Individualized Metabolic Surgery (IMS) (8), ABCD score (9), the Diabetes Remission (DiaRem) and the Advanced Diabetes Remission (Ad-DiaRem) (10). However, some patients may present complication of T2DM recurrence (11). Regarding the management of this postoperative complication, exercise is connected with the decrease of blood glucose (12) and the American Diabetes Association (ADA) advocates for individualized eating plans in nutrition therapy (13). Currently, there is no standardized pharmaceutical treatment for managing recurring T2DM, but emerging data may assist physicians in selecting appropriate medications (14). Additionally, conversional surgery was found to markedly control blood glucose with higher risks of complications compared to the primary surgery.

Our study provides a narrative review of the factors that can predict weight loss, remission of diabetes, and strategies for managing the post-BS RWG and diabetes recurrence.

## 2 Predictors of postoperative outcomes

### 2.1 Predictors of postoperative weight loss

The extent of weight loss variation is likely influenced more by biological elements that affect neuro-endocrine processes, as well as psychological factors that impact eating habits (15). It is probable that a sophisticated interaction between biological and mental processes plays a significant role (15). An increase in age has often been identified as a factor that predicts a reduced weight loss outcome post-surgery (16–19). Elder people often exhibit a decreased basal metabolic rate coupled with a reduction in physical activity (20). Additionally, as age advances, there is a growing challenge to alter established dietary and lifestyle habits, which leads to a less significant reduction in weight (21). A higher starting BMI typically correlates with a more substantial absolute weight loss (22). However, when weight loss is measured relatively, those with a higher initial BMI may experience a smaller percentage reduction (23–26). Specifically, patients with T2DM tend to achieve less weight loss (17, 23).

A majority of research on preoperative weight loss has yielded either negative or inconclusive findings. In a randomized controlled trial, Kalarchian et al. (27) compared patients who underwent a 6-month behavioral lifestyle intervention with those who received standard pre-surgical care. They found no significant discrepancy in post-operative weight reduction between the two groups. Similarly, Krimpuri et al. (28) have noted that the predictive power of pre-operative weight loss diminishes gradually by the one-year mark. Several retrospective studies have also failed to find compelling evidence that pre-operative weight loss is reliable for predicting post-operative weight loss (29–31). The American Society for Metabolic and Bariatric Surgery (ASMBS) has concluded that few medical evidence can support the notion that preoperative weight loss offers any advantage in terms of bariatric surgery outcomes (32). Meanwhile, Mocanu et al. (33) supposed that prroperative weight loss is needed since they verified that a reduction in weight prior to surgery correlates with better chances of survival within 30 days and a decreased likelihood of postoperative leaks. Tolvanen et al. (34) discovered that individuals who have made efforts to lose weight demonstrated enhanced cognitive restraint in their dietary habits which highlights the critical need for pharmacological and psychological assessments before BS.

### 2.2 Predictors of postoperative T2DM remission

T2DM remission is characterized by a spontaneous or intervention-induced return of hemoglobin A1c (HbA1c) levels to below 6.5% (or less than 48 mmol/mol), which is maintained for a minimum of 3 months without the diabetes medications (35). Effectiveness of Roux-en-Y gastric bypass (RYGB) and sleeve gastrectomy (SG) in decreasing blood sugar levels in obese patients with uncontrolled T2DM had been proven (36). The likelihood of remission can differ based on the specific surgical procedure performed. Furthermore, factors such as the duration of T2DM, pre-operative C-peptide levels as well as HbA1c levels have been identified as predictors of T2DM remission (8, 37).

Additionally, various scoring systems have been devised to estimate the likelihood of diabetes remission for individual patients. Aminian et al. (8) utilized a dataset comprising T2DM patients who accepted RYGB and SG procedures to create a nomogram that generated an Individualized Metabolic Surgery (IMS) score which was used to evaluate the effectiveness of different surgical techniques in achieving diabetes remission rates across various stages of T2DM severity. Lee et al. (9) introduced the Diabetes Surgery Score, also known as the ABCD score, which takes the age, C-peptide levels, BMI, and T2DM duration of patients into account. The ABCD score demonstrated prominent specificity and accurant predictivity, while it only targeted the Asian group. The Diabetes Remission (DiaRem) score incorporated preoperative clinical variables to predict the probability of T2DM remission over a five-year period. However, Aron-Wisnewsky et al. (10) discovered that the DiaRem score had limited predictive power for lower scores. As a result, the DiaBetter score was introduced, incorporating factors such as T2DM duration and glycated hemoglobin levels. Both the DiaBetter and DiaRem scores were found to have comparable predictive value for two-year T2DM remission rates following both RYGB and SG procedures (38). Ultimately, the DiaRem score’s precision and predictive capabilities were enhanced by taking into account the duration of T2DM and the dosage of hypoglycemic medications used. This led to the development of the Advanced Diabetes Remission (Ad-DiaRem) score (10). Within a cohort of Israeli individuals with five-year post-operative diabetes status data, the Ad-DiaRem score demonstrated a slight improvement over the DiaRem score in the prediction of long-term T2DM remission following RYGB surgery (39). As seen above, a highly predictive and accurate diabetes scoring system is important in predicting remission of type 2 diabetes after bariatric surgery.

## 3 Management of RWG

### 3.1 RWG after BS

In the latest meeting held by International Federation for the Surgery of Obesity and Metabolic Disorders (IFSO) (40), an agreement was established to utilize the term “recurrent weight gain (RWG)” for individuals who undergo substantial weight increase following their initial weight reduction post-surgery. This term is defined as a weight gain exceeding 30% or an exacerbation of an obesity-related complication that was a pivotal reason for undergoing surgery. Given the different efficacy of each BS procedures (41, 42) and variable effects in different patient groups, these criteria should be personalized, complemented by the expertise of clinical judgment.

### 3.2 Behavioral interventions

Empirical studies endorse the significance of MVPA, in curbing post-bariatric surgery weight regain (43). Notably, the numerous evidences are based on objective daily assessments of patients’ MVPA, which minimizes the bias associated with self-reported activity levels. As highlighted in a previous review (44), it is essential for future research to detail exercise adherence rates to guide the formulation of effective exercise programs. Consequently, interventions should focus on imparting behavioral strategies—such as self-monitoring of exercise, setting achievable goals, and scheduling exercise routines—to ensure the long-term maintenance of physical activity habits (45).

Ongoing and regular consultation with a dietitian specializing in BS is linked to enhanced outcomes in terms of weight loss success (46). Continued dietary guidance post-surgery is highly advantageous for the majority of patients (47). Patients’ capacity to digest solid foods is restricted, which calls for a gradual dietary progression from liquids to solids (48). The primary objective of dietary counseling following BS is to ensure an adequate intake of high-quality protein and recommended daily protein intake ranges from 60 to 120 grams, contingent upon the specific surgical procedure performed (49). Furthermore, it is crucial to ensure patients comply with the prescribed regimen of vitamin and mineral supplements (50). The dietary guidelines for post-BS recommends such as restricting meal portion sizes to 125 grams every 30 min and choosing foods that are rich in protein and high in fiber. This includes a variety of options like eggs, poultry, fruits, lean meats, fish, vegetables, low-fat dairy products, legumes, and whole grains (51).

### 3.3 Anti-obesity medicine

Anti-obesity medicine (AOM) options that are currently approved by the US Food and Drug Administration (FDA) include liraglutide, phentermine, phentermine/topiramate extended release (ER), naltrexone sustained release (SR) /bupropion sustained release (SR) and orlistat (52).

**Figure 1:**
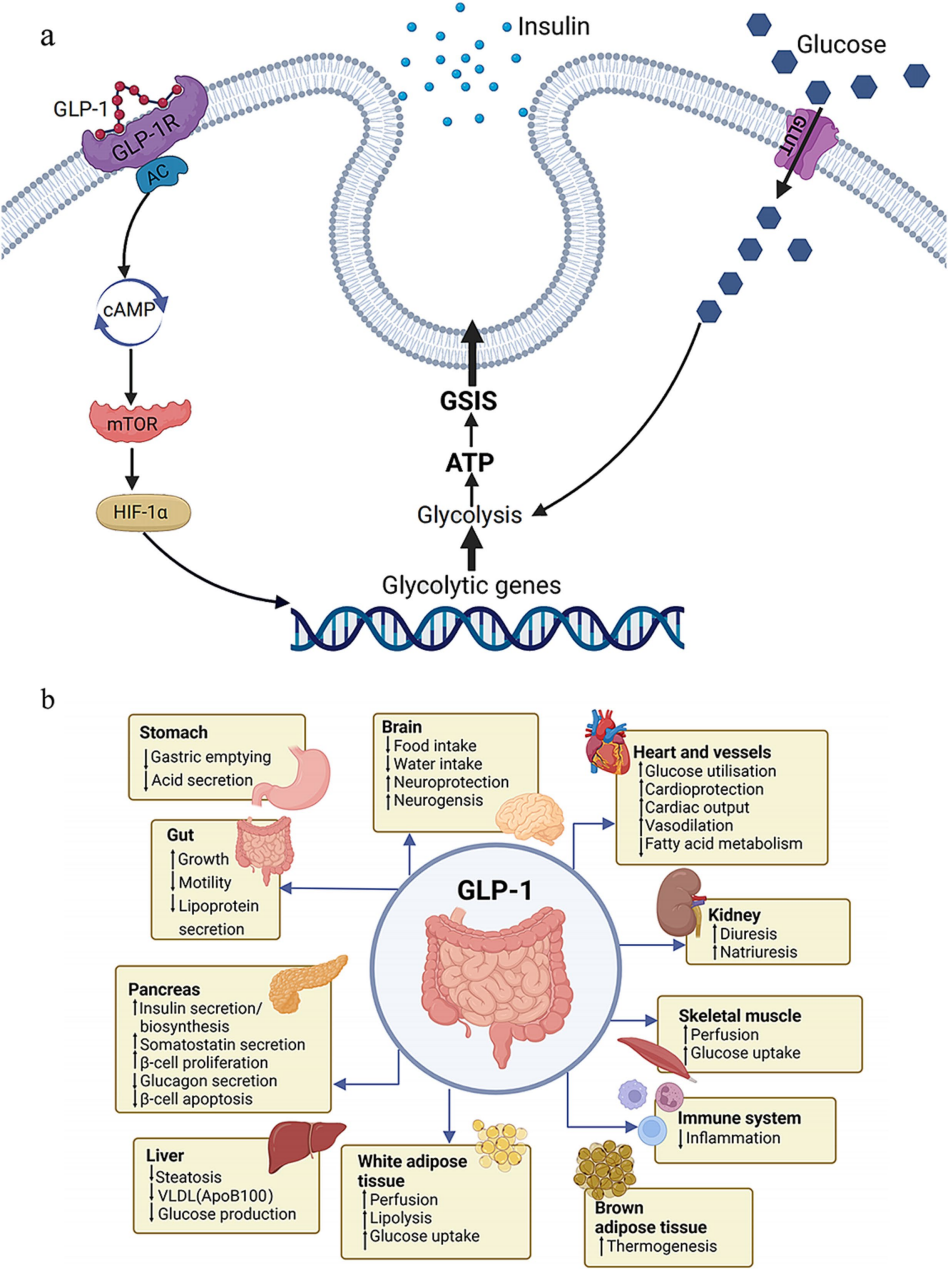
Function of GLP-1. **(A)** Molecular mechanism of GLP-1. **(B)** Effects of GLP-1 on the body. GLP-1, glucagon-like peptide-1; glucose-stimulated insulin secretion.

Liraglutide, an injectable glucagon-like peptide-1 (GLP-1) receptor agonist, has received FDA approval as an AOM agent at a dosage of 3.0 mg. It is believed to control appetite via both peripheral and central nervous system pathways and has demonstrated efficacy in bariatric surgery patients with RWG (53). Furthermore, preliminary evidence hints that GLP-1 agonists might offer a therapeutic advantage in addressing hypoglycemia (54). In addition to liraglutide, another, semaglutide, a GLP-1 agonist approved for T2DM treatment, has shown efficacy in promoting weight loss (55) and potentially surpassing liraglutide (56). The mechanism of GLP-1 (Figure 1A) and the effects of GLP-1 on the body (Figure 1B) are illustrated in Figure 1. Phentermine functions as a sympathomimetic amine, which stimulates the release of catecholamines in the hypothalamus, resulting in the suppression of appetite. When administered as a standalone treatment or in conjunction with topiramate, it has been demonstrated a reduced RWG (57) and facilitated weight reduction in cases where the outcomes of BS have been suboptimal (58). Topiramate, a medication primarily used to treat epilepsy and migraines, has not been officially approved for monotherapy in obesity treatment. However, it is frequently prescribed off-label due to its appetite-suppressing properties. It has been utilized both as a solo treatment and in combination with phentermine, as well as for the management of binge eating disorder (59). Notably, topiramate has turned into a highly effective option for managing weight regain in a subset of postoperative patients in a specific study (60). Naltrexone SR/Bupropion SR represents another FDA-approved dual-drug AOM. Each of these medications targets the central nervous system (CNS) reward pathways, and there is a theorized synergistic impact on human appetite regulation. This effect is suggested by animal studies, which propose that the combination acts on the specific receptor to enhance gorged feelings and prevent inhibited feedback. A number of randomized, double-blind, placebo-controlled trials have substantiated the efficacy of this medication pairing in combating RWG (61). Orlistat functions as a lipase inhibitor, which leads to the reduced absorption of 25–30% of the dietary fat consumed through the gastrointestinal tract (62). Current therapeutic options are reviewed in Table 1.

**Table 1:** Overview of anti-obesity medicine and the pertinent clinical factors to consider.

Drug	Use consideration	Weight loss (drug/placebo)	Slide effects
Liraglutide (117)	3.0 mg, OD, subcutaneous injection	−8%/−2.6%	Nausea/vomiting, diarrhea, constipation, pancreatitis, gallstone
Semaglutide (118)	2.4 mg, once weekly, subcutaneous injection	−14.9%/−2.4%	Nausea/vomiting, diarrhea, constipation
Phentermine (119)	15–30 mg, OD, oral	−6.6 to −7.4%/−1.7% (dose- dependent)	Palpitations, elevated blood pressure
Phentermine/topiramate ER (120)	15 mg/92 mg, OD, oral	−7.8% to −9.3%/−1.2% (dose-dependent)	Depression, suicidal ideation, memory loss, birth defects, cardiovascular events
Naltrexone SR/bupropion SR (61)	32 mg/360 mg, BID, oral	−5.0 to −6.1%/−1.3% (dose-dependent)	Seizures, palpitations, transient blood pressure elevations
Orlistat (121)	120 mg TID, oral	−10.2%/−6.1%	Liver injury, gastrointestinal symptoms

### 3.4 Endoscopic interventions

Endoscopic sleeve gastroplasty (ESG) involved creating a two-row plication, effectively reducing the size beginning from the gastroesophageal joint to the prepyloric antrum by forming a narrow sleeve-like structure (63). The first instance of a revisional ESG following SG was documented by Sharaiha and colleagues, resulting in 9-kilogram decrease (64). Across a retrospective investigation of five individuals who received a revisional ESG due to an enlarged gastric sleeve, a consistent TWL ranging from 6.7 to 17.2% was noted at the 12-month mark (65). A subsequent report detailed the revisional ESG as a “sleeve-in-sleeve” process, which involved creating additional applications in the stomach based on a special approach. The patient in this case experienced a favorable post-procedure outcome, with a weight loss of 7 kilograms, equating to an 8% TWL, reported at the three-month follow-up (66).

Transoral Outlet Reduction (TORe) after RYGB operates by constricting the gastrojejunal anastomosis (GJA) diameter with the aid of endoscopic tools and platforms that are commercially accessible. The TORe procedure diminishes the GJA’s size, thereby facilitating weight loss through a mechanical limitation that curtails hunger and enhances satiety (67). The execution of TORe can be varied, encompassing full-thickness endoscopic suturing, plications, and hybrid techniques that may include the ablation or resection of the GJA’s mucosal layer (68). The follow-up results indicated that patients who underwent TORe achieved a 3.5% total weight loss (TWL), a statistically significant improvement over the 0.4% TWL observed in the control group that received a sham procedure (*p* = 0.02) after 1 year (69). Subsequent advancements in the TORe approach have been made to boost its effectiveness (70). Argon Plasma Coagulation (APC) is a noncontact method of electrocoagulation, leading to a gradual reduction in diameter (71). The use of APC in the context of the GJA was primarily showcased in 2006 as a supplementary step during the standard TORe flow. Patients who received APC prior to suturing exhibited greater weight loss compared to those who underwent suturing without this preliminary step (72).

Restorative obesity surgery endoluminal (ROSE) is an alternative process to tackle RWG after RYGB (71). A multicenter registry reported on the outcomes of a cohort of patients who employed a non-invasive revision method to reduce the dimensions of their stoma and pouch. The study demonstrated that, at 6 months post-procedure (with data from 96 patients), there was an average weight reduction equivalent to 32% of the weight regained from the lowest weight point (73).

### 3.5 Conversional surgery

#### 3.5.1 Conversional surgery after SG

**Figure 2:**
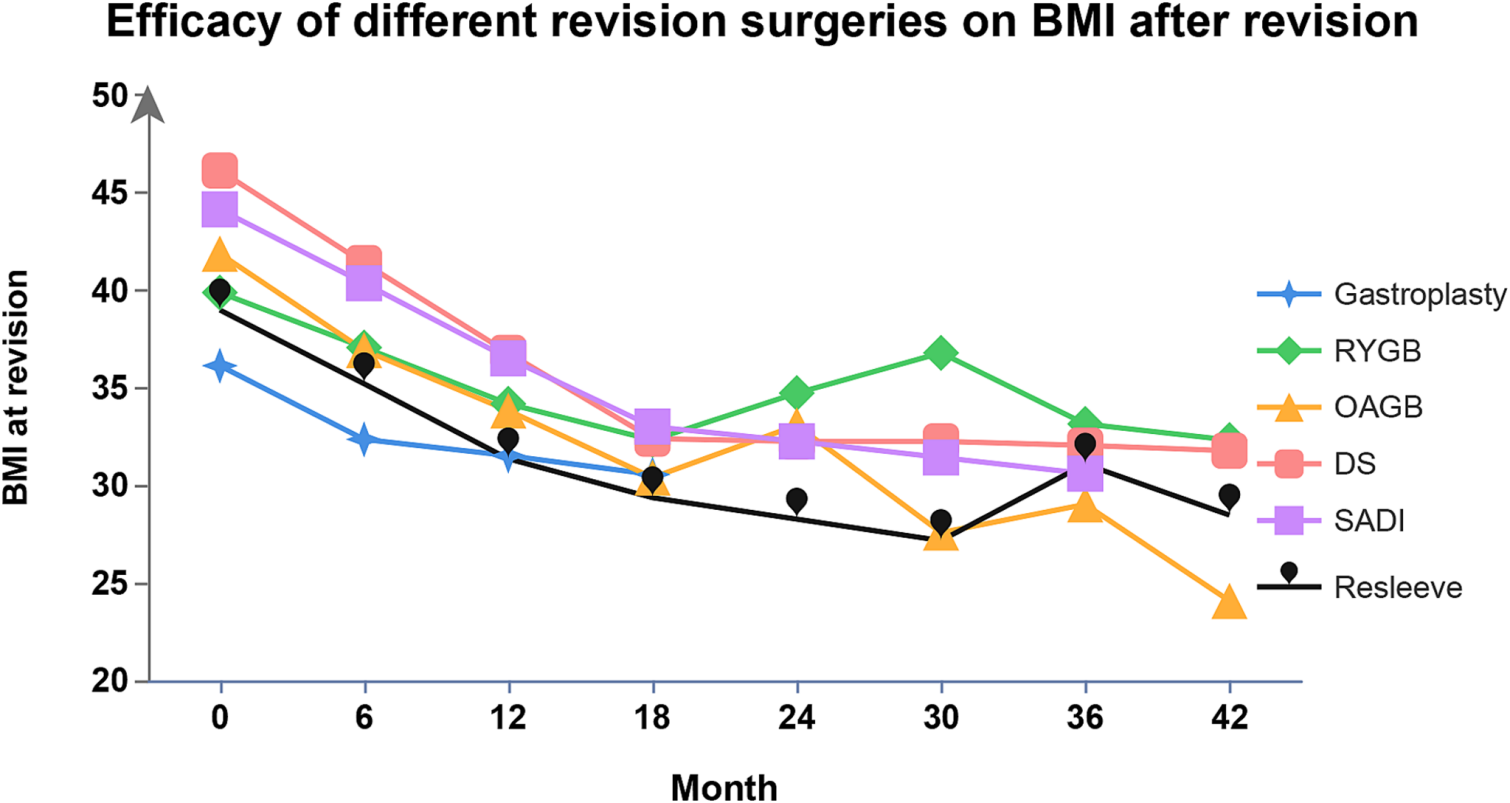
Efficacy of different conversional surgeries after SG on BMI. BMI, body mass index; SG, sleeve gastrectomy; RYGB, Roux-en-Y gastric bypass; OAGB, one-anastomosis gastric bypass; SADI, single-anastomosis duodeno-ileal bypass; DS, duodenal switch.

Conversional surgeries for RWG after SG include endoscopic sleeve gastroplasty (ESG), re-sleeve gastrectomy (RSG), RYGB, one-anastomosis gastric bypass (OAGB), single-anastomosis duodeno-ileal bypass (SADI), and duodenal switch (DS) (74). Efficacy of different conversional surgeries on BMI during follow-up are showed in Figure 2.

ESG is a non-invasive, incisionless procedure that reshapes the stomach by applying full-thickness sutures, thereby decreasing its capacity and slowing down gastric emptying (75). An observational study conducted by Sharaiha and colleagues (76) has demonstrated that ESG can lead to positive metabolic changes and improvements in obesity-related comorbidities. In cases where RWG is experienced following a SG, RSG may be considered, particularly if the stomach’s dilation exceeds 4 cm in diameter (77). RSG involves the reshaping of the remaining stomach volume. It has been suggested under circumstances where the stomach volume, as measured by a gastroscanner, surpasses 250 cc, as proposed by a French research team in 2014 (78). However, due to the high rates of postoperative gastroesophageal reflux disease (GERD), RSG is typically recommended only for select individuals with a significantly excessed the gastric fundus or antrum (79). A conversional OAGB involves the construction of a gastric pouch and the creation of a gastrojejunal anastomosis with a relatively broadened biliary limb (80). The absence of a jejuno-jejunal anastomosis in OAGB reduces the potential for future complications (81), and this procedure is also believed to decrease operative time as it necessitates the formation of only one anastomosis (82). The SADI-S was devised as a streamlined version of the biliopancreatic diversion/duodenal switch, aiming to reduce operating time and postoperative complications while retaining the same principles and effectiveness (83). The separation of the ileum results in the formation of two separate segments, which are then rejoined using a surgical connection, establishing the helpful channel that facilitates digestive absorption post-conversion from SG to DS. While DS is recognized for reaching the most significant weight reduction outcomes following an unsuccessful SG, it comes with the trade-offs of a higher risk of developing complications and the complexity inherent in the surgery (84, 85).

#### 3.5.2 Conversional surgery after RYGB

**Figure 3:**
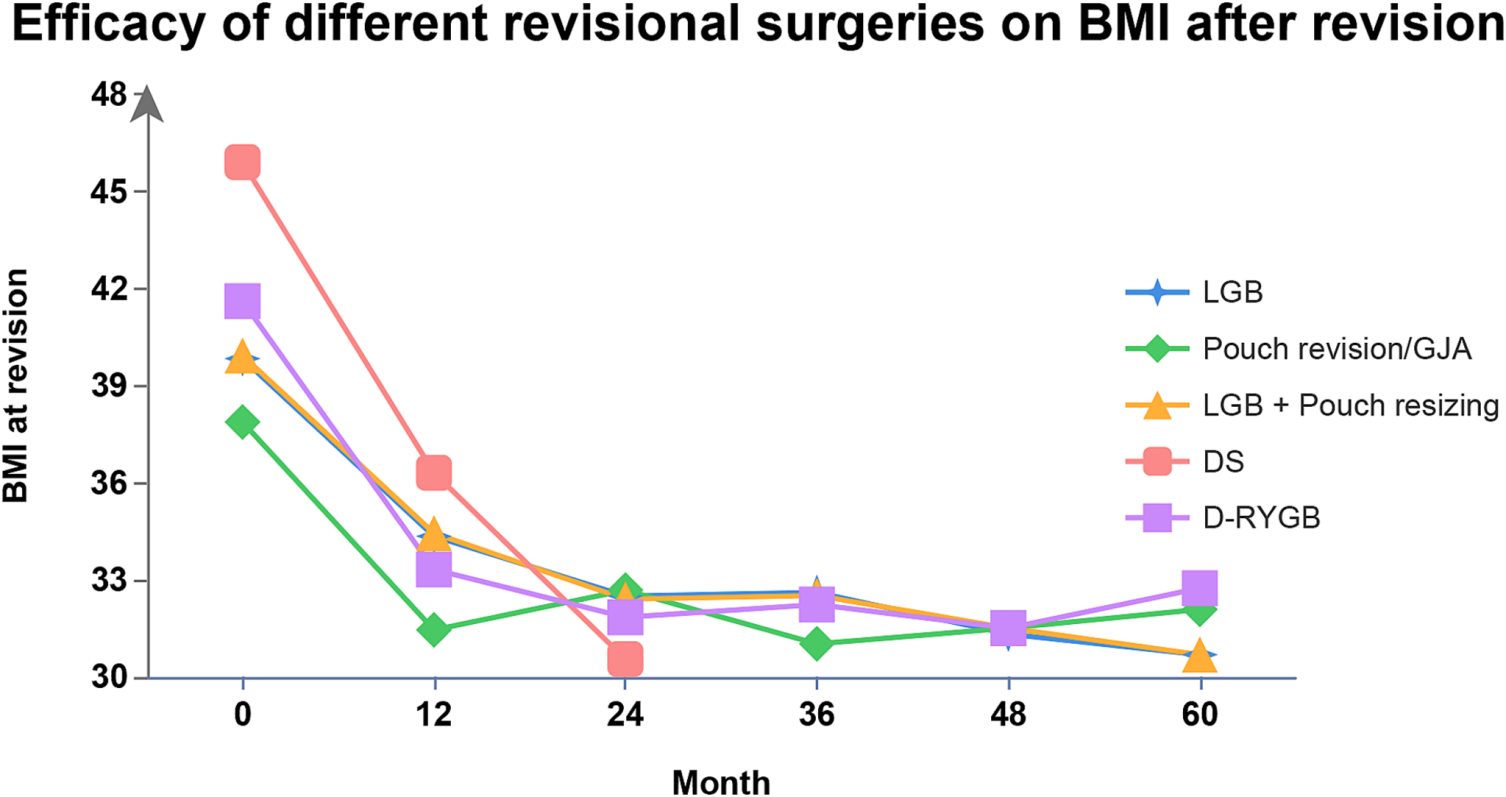
Efficacy of different conversional surgeries after RYGB on BMI. BMI, body mass index; RYGB, Roux-en-Y gastric bypass; LGB, laparoscopic gastric band; D-RYGB, distalization- Roux-en-Y gastric bypass; GJA, gastrojejunal anastomosis; DS, duodenal switch.

Conversional surgeries after RYGB include the conversion of the GJA and/or pouch, gastric band around the upper pouch with laparoscope (LGB), a band with laparoscope combined with pouch resizing, distalization-RYGB (D-RYGB) and a duodenal switch (DS). Franken et al. (86) estimated the function and safety of those clinical techniques following RYGB for RWG. Efficacy of various conversional operations on BMI in the follow-up assay is shown in Figure 3.

GJA or pouch conversion can be performed by either reshaping these structures or by removing part of the GJA followed by a reconstruction (87). LGB is a restrictive procedure that involves placing an artificial band near the gastrojejunostomy (88). DS is a more complex operation that includes creating a partial sleeve gastrectomy while keeping the pylorus intact, forming a Roux limb, extending the biliopancreatic limb, and establishing a short useful channel (89). D-RYGB is achieved by reducing the channel length, thereby enhancing the malabsorptive effect of the RYGB. There are two main methods of distalization: either by reconfiguring the Roux limb (Type 1) or the Y limb (Type 2). Shin et al. (90) suggested that an optimal total length of the alimentary limb should be around 300 centimeters to minimize the risk of malnutrition and reduce the occurrence of diarrhea, thereby improving the overall quality of life for patients undergoing these complex conversional procedures.

## 4 Management of T2DM recurrence

### 4.1 T2DM recurrence after BS

BS is typically linked to substantial enhancements or even the remission of diseases related to obesity, with a particularly notable impact on T2DM. T2DM is a multifaceted hormonal and metabolic condition characterized by varying levels of insulin resistance and impairment of the pancreatic β-cells (91). In the Swedish Obese Subjects (SOS) trial, a significant proportion of participants with T2DM at the outset—72%—were observed to be in remission after a 2-year post-bariatric surgery follow-up period. However, a noteworthy 50% of these individuals saw a return of T2DM symptoms by the 10-year check-up (92). The STAMPEDE was a randomized trial evaluating and comparing RYGB versus sleeve gastrectomy SG in obese patients with T2DM. The trial’s data indicated a decline in the remission rates of T2DM; after RYGB, the percentage dropped from 78% in the initial year to 45% at the 5th year, and following SG, it decreased from 51 to 25% over the same period (93). Definition of baseline T2DM and the recurrence within a 10- or 15-year period is defined as an HbA1c level of 48 mmol/mol or higher, a blood glucose level of 6.1 mmol/L or above (plasma glucose of 7 mmol/L or above), or the use of T2DM medications (11) (Table 2).

**Table 2:** Physical interventions which ADA recommends and its corresponding frequency and targeted individuals.

Physical intervention	Frequency	Targeted individuals
Aerobic activities of a moderate to intense nature	60 min/day or more	Youth with T2DM
Intense muscle-fortifying and bone-strengthening exercises	At least 3 days/week	Youth with T2DM
Aerobic exercises that are either of moderate or vigorous intensity	A minimum of 150 min per day of physical activity, distributed over at least 3 days per week, ensuring that there are no more than two consecutive days without engaging in any form of activity	Most adults with T2DM
Strength training exercises performed on alternate days	2–3 sessions/week	Most adults with T2DM
Exercises aimed at enhancing flexibility and improving balance	2–3 times/week	Older adults with T2DM

The ADA holds the stance that there is not a uniform dietary approach suitable for all individuals with diabetes. Traditionally, the ADA has endorsed a personalized dietary plan developed through a collaborative effort tailored to the specific necessities and preferences of the diabetes populations (13). The following eating patterns are listed in Table 3. Various dietary patterns for specific individuals have been shown to achieve varying levels of health benefits as evidence accumulates. Healthcare providers should concentrate on the fundamental elements shared across these patterns, which include reducing the consumption of added sugars, prioritizing the intake of nonstarchy vegetables and refined grains, and opting for whole foods over their highly processed counterparts whenever feasible (94).

**Table 3:** Eating patterns recommended.

Category of eating pattern	Description
Mediterranean-style (122)	Highlights the consumption of plant-derived foods and seafood; designates olive oil as the primary source of dietary fats; includes dairy in moderate to small quantities; allows for a typical intake of less than four eggs per week; limits the intake of red meat to infrequent and small portions; permits wine in measured amounts; and discourages the regular use of concentrated sugars or honey
Vegetarian or vegan (123)	Vegetarian diets are typically categorized into two primary types: vegan diets, which exclude all animal flesh and by-products, and vegetarian diets, which forgo animal flesh but may include eggs and/or dairy products. A vegetarian dietary pattern is defined by a lower intake of saturated fats and cholesterol, coupled with a higher intake of fruits, vegetables, whole grains, nuts, soy products, fiber, and plant-derived compounds
Low-carbohydrate (124)	Concentrates on consuming foods that are rich in protein, such as meats, poultry, fish, shellfish, eggs, cheese, as well as nuts and seeds. Emphasizes the intake of healthy fats from sources like oils, butter, olives, and avocados, along with low-carbohydrate vegetables including salad greens, cucumbers, broccoli, and summer squash. While most plans permit some carbohydrates in the form of fruits, particularly berries, and higher carbohydrate vegetables, they generally discourage the consumption of sugary foods and grain-based products like pasta, rice, and bread
Low-fat (125)	Emphasizes the intake of vegetables, fruits, and starchy items such as bread, crackers, pasta, whole grains, and starchy vegetables. It also includes lean protein sources like legumes and suggests the use of low-fat dairy options. This dietary approach is characterized by a total fat intake that does not exceed 30% of total daily calories and a saturated fat intake that is capped at 10% or less
Dietary Approaches to Stop Hypertension (DASH) (126)	Highlights the consumption of fruits, vegetables, and low-fat dairy, alongside whole grains, poultry, fish, and nuts, while limiting intake of saturated fats, red meats, desserts, and sugary drinks. The most effective version of the DASH diet also incorporates a reduced sodium content

### 4.2 Hypoglycemic agents

**Figure 4:**
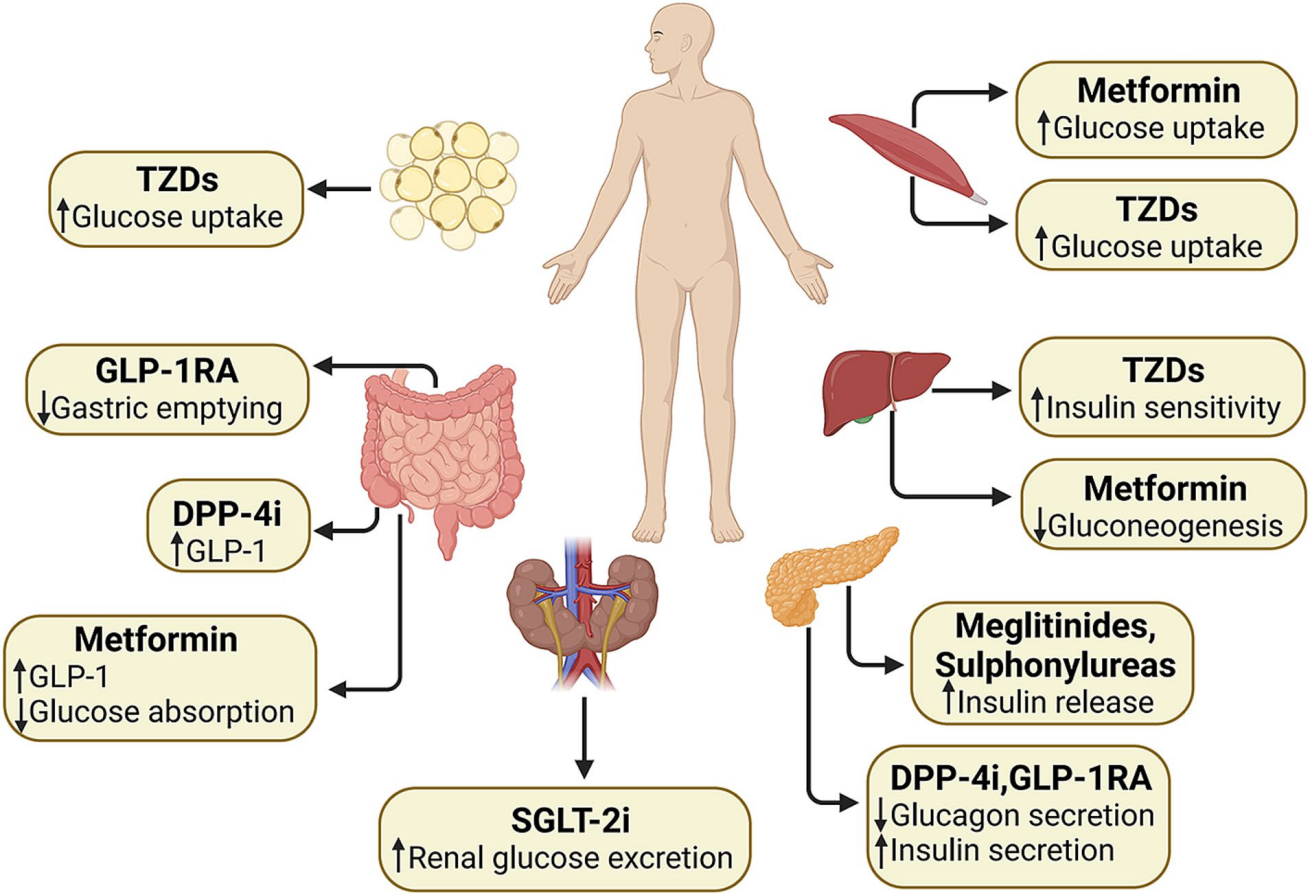
Effects of different types of hypoglycemic agents on the body. GLP-1, glucagon-like peptide-1; GLP-1 RA, glucagon-like peptide-1 receptor agonists; DPP-4i, dipeptidyl peptidase-4 inhibitors; SGLT-2i, sodium–glucose cotransporter 2 inhibitors; TZDs, thiazolidinediones.

The ADA lists first-line hypoglycemic agents in its latest guidelines, including GLP-1 receptor agonists (GLP-1RA), dipeptidyl peptidase 4 (DPP-4) inhibitors (DPP-4i), sodium–glucose cotransporter 2 (SGLT-2) inhibitors (SGLT-2i), metformin, thiazolidinediones (TZDs), sulfonylureas and insulin (95). GLP-1 RAs target the pancreas to stimulate the release of insulin and curb the production of glucagon with the function within the gastrointestinal tract to slow down the process of gastric emptying (96). DPP-4i elevate endogenous incretin levels by inhibiting the activity of DPP-4 (96). SGLT-2i decrease renal glucose reabsorption (97). Metformin might target the liver to suppress gluconeogenesis and skeletal muscles to improve the utilization of glucose (98). It may also play a role in the gut by increasing the levels of GLP-1 (99). TZDs enhance insulin sensitivity in the skeletal muscles, adipose tissue, and liver. Sulfonylureas stimulate the pancreas to augment the secretion of insulin (100). Figure 4 shows the mechanism and target organ of hypoglycemic agents. Factors that are specific to the individual and influence the selection of treatment encompass personalized blood sugar targets (101), the person’s susceptibility to hypoglycemia, and their medical history or risk factors associated with cardiovascular, renal, hepatic, and other comorbidities and complications related to diabetes (102–104). Potential therapeutic options are reviewed in Table 4.

**Table 4:** Overview of blood glucose-lowering medications and the pertinent clinical factors to consider.

Glucose lowering agent*	Efficacy (127, 128)	Body weight (127, 128)	Progression on DKD (129–133)	Effect on MACE (134–140)	Heart failure (141–144)	Using considerations (95)	Considerations for patients following BS (95)
GLP-1 RAs	High to vitally high	Loss (intermediate to vitally high)	Benefit for renal endpoints CVOTs, driven by albuminuria: dulaglutide, liraglutide, semaglutide	Benefit: dulaglutide, liraglutide, semaglutide	Neutral	Refer to the product labels for guidance on dosage adjustments related to renal function for each specific medication Regularly assess kidney function when starting or increasing the dosage of medications in patients with compromised renal function who experience serious gastrointestinal side effects No dose adjustment for dulaglutide, liraglutide, semaglutide	Advise patients on the likelihood of gastrointestinal side effects and reassure them that these are usually short-lived; offer recommendations for dietary changes to alleviate these effects and consider a more gradual dosage adjustment for those experiencing gastrointestinal discomfort Warn patients about the possibility of ileus (semaglutide) If symptoms of gallstones or cholecystitis arise, assess for gallbladder disease
SGLT-2 inhibitors	Intermediate to high	Loss (intermediate)	Benefit: canagliflozin, empagliflozin, dapagliflozin	Benefit: canagliflozin, empagliflozin	Benefit: canagliflozin, empagliflozin, dapagliflozin, ertugliflozin (141–144)	Refer to the product labels for guidance on dosage adjustments related to renal function for each specific medication The efficacy of SGLT2 inhibitors in reducing blood glucose levels is diminished when the estimated glomerular filtration rate (eGFR) is low	There is an elevated risk of euglycemic diabetic ketoacidosis (eDKA) during the perioperative period, as well as an increased susceptibility to dehydration and vitamin D deficiency Heightened vulnerability to genital mycotic infections It is crucial to closely monitor the patient’s volume status and blood pressure, and make necessary adjustments to other medications that could affect volume status
DPP-4 inhibitors	Intermediate	Neutral	Neutral	Neutral	Neutral (potential risk, saxagliptin) (145)	Renal dose adjustment required (sitagliptin, saxagliptin, alogliptin) No dose adjustment required for linagliptin	Instances of pancreatitis have been documented in clinical studies, yet a definitive causal relationship has not been confirmed. Should there be any suspicion of pancreatitis, the medication should be discontinued immediately
Metformin	High	Neutral (potential for modest loss)	Neutral	Potential benefit	Neutral	Contraindicated with eGFR <30 mL/min per 1.73 m^2^	GI side effects common due to increased bioavailability; to mitigate it, consider slow dose titration, administration with food and extended-release formulations Increased risk of Vit B12 deficiency; monitor regularly
Thiazolidinediones	High	Gain	Neutral	Potential benefit: pioglitazone	Increased risk (146)	No dose adjustment required Typically, their use is not advised in cases of renal impairment due to the risk of fluid retention	Congestive heart failure (pioglitazone, rosiglitazone) Risk of bone fractures Fluid retention (heart failure; edema)
Sulfonylureas	High	Gain	Neutral	Neutral	Neutral	Glipizide and glimepiride should be started at a lower dose to minimize the risk of hypoglycemia Glyburide: generally not recommended in chronic kidney disease	Use with caution in individuals at risk for hypoglycemia
Insulin	High to vitally high	Gain	Neutral	Neutral	Neutral	Lower insulin doses required with a decrease in eGFR; titrate per clinical response	Higher risk of hypoglycemia with human insulin (NPH or premixed formulations) vs. analogs Monitor injection site reactions

DKA, diabetic ketoacidosis; DKD, diabetic kidney disease; DPP-4, dipeptidyl peptidase 4; eGFR, estimated glomerular filtration rate; GI, gas-trointestinal; MACE, major adverse cardiovascular events; SGLT2, sodium–glucose cotransporter 2; * For agent-specific dosing recommendations, please refer to manufacturers’ prescribing information (127, 128).

### 4.3 Conversional surgeries

Yan et al. (105) has evaluated the influence of conversional surgery on T2DM. Aleassa EM et al. corroborated findings that the overall improvement in T2DM can vary from 65 to 100%, contingent upon the specific indices and types of reconstructive surgery performed (106). Below, we will review types of conversional operations undergone: vertical banded gastroplasty (VBG) to Roux-en-Y gastric bypass (RYGB), adjustable gastric banding (AGB) conversions, sleeve gastrectomy (SG) conversions, and conversion of pouch/stoma after RYGB.

Challenges such as band erosion, dysphagia, and staple line failure have diminished the use of VBG. However, due to the anatomical changes induced by the procedure, converting VBG to RYGB has been shown to offer metabolic benefits for individuals with T2DM (107–109). Gagné et al. (110) examined data from patients under this conversion from July 1999 to April 2010 and discovered that T2DM improved or resolved in 90% of cases. Sarhan et al. (111) reviewed records of 102 patients who had a conversional RYGB following an unsuccessful VBG from April 2014 to January 2018, noting a T2DM improvement rate of 75.7% with complete remission and a 24.3% partial remission. Ngiam et al. (112) demonstrated the effectiveness of these conversional surgeries in resolving diabetes compared to AGB. Vidal et al. (113) reported comparable resolution rates for T2DM after SG and RYGB (51.4% vs. 62.0%) at the four-month mark post-surgery. Yeung et al. (114) observed little significant discrepancy in medication reduction for diabetes and hypertension at 12 months after RYGB (33% reduction, no cessation of diabetes medication) and SG (60% reduction, 40% off diabetes medication). Lee et al. (115) divided T2DM into three assessed stages at the initial time, enabling selection of procedures from evidence-based practices. Both procedures significantly improved T2DM in mild (IMS score ≤ 25) and severe cases (IMS score > 95), but RYGB was notably more effective in intermediate cases due to its more pronounced neurohormonal impact. Conversion of pouch/stoma after RYGB in cohort demonstrated by Aleassa et al. (106) resulted in further significant weight reduction and controlling T2DM better. Rawlins et al. (116) reviewed cases from 2002 to 2009 involving the conversion of RYGB to a distal gastric bypass, revealing that patients who underwent this distalization to 100-cm distal common channel experienced improvements in diabetes management.

## 5 Conclusion

BS remains the most potent treatment for weight reduction and alleviating the T2DM. However, the postoperative outcomes can vary significantly among patients. Factors after the surgery have a more substantial influence on predicting postoperative weight loss compared to those assessed before the procedure. Despite this, there is a scarcity of holistic predictive models that anticipate weight loss outcomes post-surgery. Therefore, there is a need for scoring systems that can amalgamate various factors and accurately forecast weight loss outcomes. In this context, certain models, notably the Ad-DiaRem, demonstrate a relatively strong ability to predict the remission of T2DM following BS. It is essential to recognize that enhanced diabetes management is a significant achievement, even if it does not result in complete remission. Some patients might experience RWG and a recurrence of diabetes. Given that most postoperative behavioral factors are modifiable, proactive measures to influence postoperative eating habits and lifestyle changes are crucial. Furthermore, recommendations for behavioral interventions should be tailored to meet the specific needs of each patient. In terms of pharmaceutical treatment, healthcare providers should adhere to the same principles for dose initiation and titration as they would for patients who have not undergone surgery. The treatment should commence with the lowest possible dose, with subsequent adjustments made based on individual requirements. In cases where monotherapy proves insufficient, combination therapies can be considered. Endoscopic management, recognized for its minimally invasive nature, has predominantly been realized through standard or modified TORe in patients who have undergone RYGB. Concurrently, ESG is gaining traction as a secure and effective method for those who have had SG. However, the sustainability of these procedures is uncertain without concurrent dietary and lifestyle interventions. There is an evident need for a prospective, randomized study to evaluate this innovative technique. It is crucial to acknowledge that the advantageous outcomes of conversional surgeries are often coupled with an increased risk and complexity of complications. Therefore, referral centers should be considered the most suitable venues for conducting conversional surgeries, and stringent postoperative surveillance is imperative.

## References

[1] Blüher M. Obesity: global epidemiology and pathogenesis. Nat Rev Endocrinol. (2019) 15:288–98. doi: 10.1038/s41574-019-0176-8, PMID: 30814686

[2] Busetto L Dicker D Azran C Batterham RL Farpour-Lambert N Fried M. Obesity management task force of the European Association for the Study of obesity released "practical recommendations for the post-bariatric surgery medical management". Obes Surg. (2018) 28:2117–21. doi: 10.1007/s11695-018-3283-z, PMID: 29725979

[3] Livhits M Mercado C Yermilov I Parikh JA Dutson E Mehran A. Patient behaviors associated with weight regain after laparoscopic gastric bypass. Obes Res Clin Pract. (2011) 5:e169–266. doi: 10.1016/j.orcp.2011.03.004, PMID: 24331108

[4] Istfan NW Lipartia M Anderson WA Hess DT Apovian CM. Approach to the patient: Management of the Post-Bariatric Surgery Patient with Weight Regain. J Clin Endocrinol Metab. (2021) 106:251–63. doi: 10.1210/clinem/dgaa702, PMID: 33119080 PMC7765654

[5] Horváth L Mráz M Jude EB Haluzík M. Pharmacotherapy as an augmentation to bariatric surgery for obesity. Drugs. (2024) 84:933–52. doi: 10.1007/s40265-024-02029-0, PMID: 38970626 PMC11343883

[6] Vargas EJ Bazerbachi F Rizk M Rustagi T Acosta A Wilson EB. Transoral outlet reduction with full thickness endoscopic suturing for weight regain after gastric bypass: a large multicenter international experience and meta-analysis. Surg Endosc. (2018) 32:252–9. doi: 10.1007/s00464-017-5671-1, PMID: 28664438 PMC9707293

[7] Karmali S Brar B Shi X Sharma AM de Gara C Birch DW. Weight recidivism post-bariatric surgery: a systematic review. Obes Surg. (2013) 23:1922–33. doi: 10.1007/s11695-013-1070-4, PMID: 23996349

[8] Aminian A Brethauer SA Andalib A Nowacki AS Jimenez A Corcelles R. Individualized metabolic surgery score: procedure selection based on Diabetes severity. Ann Surg. (2017) 266:650–7. doi: 10.1097/SLA.0000000000002407, PMID: 28742680

[9] Lee WJ Hur KY Lakadawala M Kasama K Wong SK Chen SC. Predicting success of metabolic surgery: age, body mass index, C-peptide, and duration score. Surg Obes Relat Dis. (2013) 9:379–84. doi: 10.1016/j.soard.2012.07.015, PMID: 22963817

[10] Aron-Wisnewsky J Sokolovska N Liu Y Comaneshter DS Vinker S Pecht T. The advanced-DiaRem score improves prediction of diabetes remission 1 year post-Roux-en-Y gastric bypass. Diabetologia. (2017) 60:1892–902. doi: 10.1007/s00125-017-4371-7, PMID: 28733906

[11] Sjöholm K Svensson PA Taube M Jacobson P Andersson-Assarsson JC Carlsson LMS. Evaluation of prediction models for type 2 Diabetes relapse after post-bariatric surgery remission: a post hoc analysis of 15-year follow-up data from the Swedish obese subjects (SOS) study. Obes Surg. (2020) 30:3955–60. doi: 10.1007/s11695-020-04763-2, PMID: 32535782 PMC7467912

[12] Piercy KL Troiano RP Ballard RM Carlson SA Fulton JE Galuska DA. The physical activity guidelines for Americans. JAMA. (2018) 320:2020–8. doi: 10.1001/jama.2018.14854, PMID: 30418471 PMC9582631

[13] Inzucchi SE Bergenstal RM Buse JB Diamant M Ferrannini E Nauck M. Management of hyperglycemia in type 2 diabetes: a patient-centered approach: position statement of the American Diabetes Association (ADA) and the European Association for the Study of Diabetes (EASD). Diabetes Care. (2012) 35:1364–79. doi: 10.2337/dc12-0413, PMID: 22517736 PMC3357214

[14] Chee YJ Dalan R. Novel therapeutics for type 2 Diabetes mellitus-a Look at the past decade and a glimpse into the future. Biomedicines. (2024) 12:1386. doi: 10.3390/biomedicines12071386, PMID: 39061960 PMC11274090

[15] Belligoli A Bettini S Segato G Busetto L. Predicting responses to bariatric and metabolic surgery. Curr Obes Rep. (2020) 9:373–9. doi: 10.1007/s13679-020-00390-1, PMID: 32542590

[16] Busetto L Segato G De Marchi F Foletto M De Luca M Caniato D. Outcome predictors in morbidly obese recipients of an adjustable gastric band. Obes Surg. (2002) 12:83–92. doi: 10.1381/096089202321144649, PMID: 11868305

[17] Hatoum IJ Stein HK Merrifield BF Kaplan LM. Capacity for physical activity predicts weight loss after Roux-en-Y gastric bypass. Obesity (Silver Spring, MD). (2009) 17:92–9. doi: 10.1038/oby.2008.507, PMID: 18997674 PMC4226065

[18] Busetto L Angrisani L Basso N Favretti F Furbetta F Lorenzo M. Safety and efficacy of laparoscopic adjustable gastric banding in the elderly. Obesity (Silver Spring, MD). (2008) 16:334–8. doi: 10.1038/oby.2007.85, PMID: 18239641

[19] Steinbeisser M McCracken J Kharbutli B. Laparoscopic sleeve gastrectomy: preoperative weight loss and other factors as predictors of postoperative success. Obes Surg. (2017) 27:1508–13. doi: 10.1007/s11695-016-2520-6, PMID: 28050788

[20] Pannemans DL Westerterp KR. Energy expenditure, physical activity and basal metabolic rate of elderly subjects. Br J Nutr. (1995) 73:571–81. doi: 10.1079/BJN19950059, PMID: 7794872

[21] Pfefferkorn U Hort S Beluli M La Vista M Züger T. Weight loss after bariatric surgery in different age groups. Obes Surg. (2023) 33:1154–9. doi: 10.1007/s11695-023-06488-4, PMID: 36757647

[22] Larsen JK Geenen R Maas C de Wit P van Antwerpen T Brand N. Personality as a predictor of weight loss maintenance after surgery for morbid obesity. Obes Res. (2004) 12:1828–34. doi: 10.1038/oby.2004.227, PMID: 15601979

[23] Ma Y Pagoto SL Olendzki BC Hafner AR Perugini RA Mason R. Predictors of weight status following laparoscopic gastric bypass. Obes Surg. (2006) 16:1227–31. doi: 10.1381/096089206778392284, PMID: 16989709

[24] Lutfi R Torquati A Sekhar N Richards WO. Predictors of success after laparoscopic gastric bypass: a multivariate analysis of socioeconomic factors. Surg Endosc. (2006) 20:864–7. doi: 10.1007/s00464-005-0115-8, PMID: 16738971

[25] Chau WY Schmidt HJ Kouli W Davis D Wasielewski A Ballantyne GH. Patient characteristics impacting excess weight loss following laparoscopic adjustable gastric banding. Obes Surg. (2005) 15:346–50. doi: 10.1381/0960892053576811, PMID: 15826467

[26] Dixon JB Dixon ME O'Brien PE. Pre-operative predictors of weight loss at 1-year after lap-band surgery. Obes Surg. (2001) 11:200–7. doi: 10.1381/096089201321577884, PMID: 11355027

[27] Kalarchian MA Marcus MD Courcoulas AP Cheng Y Levine MD. Preoperative lifestyle intervention in bariatric surgery: a randomized clinical trial. Surg Obes Relat Dis. (2016) 12:180–7. doi: 10.1016/j.soard.2015.05.004, PMID: 26410538 PMC5041299

[28] Krimpuri RD Yokley JM Seeholzer EL Horwath EL Thomas CL Bardaro SJ. Qualifying for bariatric surgery: is preoperative weight loss a reliable predictor of postoperative weight loss? Surg Obes Relat Dis. (2018) 14:60–4. doi: 10.1016/j.soard.2017.07.012, PMID: 29287756

[29] Giordano S Victorzon M. The impact of preoperative weight loss before laparoscopic gastric bypass. Obes Surg. (2014) 24:669–74. doi: 10.1007/s11695-013-1165-y, PMID: 24357128

[30] Sherman WE Lane AE Mangieri CW Choi YU Faler BJ. Does preoperative weight change predict postoperative weight loss after laparoscopic sleeve gastrectomy? Bariatric Surg Pract Patient Care. (2015) 10:126–9. doi: 10.1089/bari.2015.0023, PMID: 26421248 PMC4575532

[31] McNickle AG Bonomo SR. Predictability of first-year weight loss in laparoscopic sleeve gastrectomy. Surg Endosc. (2017) 31:4145–9. doi: 10.1007/s00464-017-5467-3, PMID: 28281113

[32] Kim JJ Rogers AM Ballem N Schirmer B. ASMBS updated position statement on insurance mandated preoperative weight loss requirements. Surg Obes Relat Dis. (2016) 12:955–9. doi: 10.1016/j.soard.2016.04.019, PMID: 27523728

[33] Mocanu V Marcil G Dang JT Birch DW Switzer NJ Karmali S. Preoperative weight loss is linked to improved mortality and leaks following elective bariatric surgery: an analysis of 548,597 patients from 2015-2018. Surg Obes Relat Dis. (2021) 17:1846–53. doi: 10.1016/j.soard.2021.06.021, PMID: 34330621

[34] Tolvanen L Christenson A Eke H Bonn SE Lagerros YT. Weight loss history and its Association with self-esteem and eating behaviors in adolescents and young adults with obesity. Obes Facts. (2023) 16:293–300. doi: 10.1159/000529267, PMID: 36696892 PMC10331152

[35] Riddle MC Cefalu WT Evans PH Gerstein HC Nauck MA Oh WK. Consensus report: definition and interpretation of remission in type 2 Diabetes. Diabetes Care. (2021) 44:2438–44. doi: 10.2337/dci21-0034, PMID: 34462270 PMC8929179

[36] Schauer PR Kashyap SR Wolski K Brethauer SA Kirwan JP Pothier CE. Bariatric surgery versus intensive medical therapy in obese patients with diabetes. N Engl J Med. (2012) 366:1567–76. doi: 10.1056/NEJMoa1200225, PMID: 22449319 PMC3372918

[37] Brethauer SA Aminian A Romero-Talamás H Batayyah E Mackey J Kennedy L. Can diabetes be surgically cured? Long-term metabolic effects of bariatric surgery in obese patients with type 2 diabetes mellitus. Ann Surg. (2013) 258:628–636; discussion 636–637. doi: 10.1097/SLA.0b013e3182a5034b, PMID: 24018646 PMC4110959

[38] Pucci A Tymoszuk U Cheung WH Makaronidis JM Scholes S Tharakan G. Type 2 diabetes remission 2 years post Roux-en-Y gastric bypass and sleeve gastrectomy: the role of the weight loss and comparison of DiaRem and DiaBetter scores. Diabet Med. (2018) 35:360–7. doi: 10.1111/dme.13532, PMID: 29055156 PMC5836992

[39] Dicker D Golan R Aron-Wisnewsky J Zucker JD Sokolowska N Comaneshter DS. Prediction of long-term Diabetes remission after RYGB, sleeve gastrectomy, and adjustable gastric banding using DiaRem and advanced-DiaRem scores. Obes Surg. (2019) 29:796–804. doi: 10.1007/s11695-018-3583-3, PMID: 30467708

[40] Haddad A Suter M Greve JW Shikora S Prager G Dayyeh BA. Therapeutic options for recurrence of weight and obesity related complications after metabolic and bariatric surgery: an IFSO position statement. Obes Surg. (2024) 34:3944–62. doi: 10.1007/s11695-024-07489-7, PMID: 39400870

[41] Peterli R Wölnerhanssen BK Peters T Vetter D Kröll D Borbély Y. Effect of laparoscopic sleeve gastrectomy vs laparoscopic Roux-en-Y gastric bypass on weight loss in patients with morbid obesity: the SM-BOSS randomized clinical trial. JAMA. (2018) 319:255–65. doi: 10.1001/jama.2017.20897, PMID: 29340679 PMC5833546

[42] Salminen P Helmiö M Ovaska J Juuti A Leivonen M Peromaa-Haavisto P. Effect of laparoscopic sleeve gastrectomy vs laparoscopic Roux-en-Y gastric bypass on weight loss at 5 years among patients with morbid obesity: the SLEEVEPASS randomized clinical trial. JAMA. (2018) 319:241–54. doi: 10.1001/jama.2017.20313, PMID: 29340676 PMC5833550

[43] Josbeno DA Kalarchian M Sparto PJ Otto AD Jakicic JM. Physical activity and physical function in individuals post-bariatric surgery. Obes Surg. (2011) 21:1243–9. doi: 10.1007/s11695-010-0327-4, PMID: 21153567 PMC4887858

[44] Baillot A St-Pierre M Bernard P Burkhardt L Chorfi W Oppert JM. Exercise and bariatric surgery: a systematic review and meta-analysis of the feasibility and acceptability of exercise and controlled trial methods. Obes Rev. (2022) 23:e13480. doi: 10.1111/obr.13480, PMID: 35695385

[45] Bond DS Thomas JG Vithiananthan S Unick J Webster J Roye GD. Intervention-related increases in preoperative physical activity are maintained 6-months after bariatric surgery: results from the bari-active trial. Int J Obes. (2017) 41:467–70. doi: 10.1038/ijo.2016.237, PMID: 28025574 PMC5340609

[46] Freire RH Borges MC Alvarez-Leite JI Correia MITD. Food quality, physical activity, and nutritional follow-up as determinant of weight regain after Roux-en-Y gastric bypass. Nutrition (Burbank, Los Angeles County, Calif). (2012) 28:53–8. doi: 10.1016/j.nut.2011.01.011, PMID: 21885246

[47] Rusch MD Andris D. Maladaptive eating patterns after weight-loss surgery. Nutr Clin Pract. (2007) 22:41–9. doi: 10.1177/011542650702200141, PMID: 17242453

[48] Bosnic G. Nutritional requirements after bariatric surgery. Crit Care Nurs Clin North Am. (2014) 26:255–62. doi: 10.1016/j.ccell.2014.02.002, PMID: 24878210

[49] Mechanick JI Youdim A Jones DB Timothy Garvey W Hurley DL Molly McMahon M. Clinical practice guidelines for the perioperative nutritional, metabolic, and nonsurgical support of the bariatric surgery patient—2013 update: cosponsored by American Association of Clinical Endocrinologists, the Obesity Society, and American Society for Metabolic & bariatric surgery. Surg Obes Relat Dis. (2013) 9:159–91. doi: 10.1016/j.soard.2012.12.010, PMID: 23537696

[50] Magallares A Schomerus G. Mental and physical health-related quality of life in obese patients before and after bariatric surgery: a meta-analysis. Psychol Health Med. (2015) 20:165–76. doi: 10.1080/13548506.2014.963627, PMID: 25258028

[51] Parkes E. Nutritional management of patients after bariatric surgery. Am J Med Sci. (2006) 331:207–13. doi: 10.1097/00000441-200604000-00007, PMID: 16617236

[52] Srivastava G Apovian CM. Current pharmacotherapy for obesity. Nat Rev Endocrinol. (2018) 14:12–24. doi: 10.1038/nrendo.2017.122, PMID: 29027993

[53] Miras AD Pérez-Pevida B Aldhwayan M Kamocka A McGlone ER Al-Najim W. Adjunctive liraglutide treatment in patients with persistent or recurrent type 2 diabetes after metabolic surgery (GRAVITAS): a randomised, double-blind, placebo-controlled trial. Lancet Diab Endocrinol. (2019) 7:549–59. doi: 10.1016/S2213-8587(19)30157-3, PMID: 31174993

[54] Abrahamsson N Engström BE Sundbom M Karlsson FA. GLP1 analogs as treatment of postprandial hypoglycemia following gastric bypass surgery: a potential new indication? Eur J Endocrinol. (2013) 169:885–9. doi: 10.1530/EJE-13-0504, PMID: 24086087

[55] Ahrén B Atkin SL Charpentier G Warren ML Wilding JPH Birch S. Semaglutide induces weight loss in subjects with type 2 diabetes regardless of baseline BMI or gastrointestinal adverse events in the SUSTAIN 1 to 5 trials. Diabetes Obes Metab. (2018) 20:2210–9. doi: 10.1111/dom.13353, PMID: 29766634 PMC6099440

[56] Murvelashvili N Xie L Schellinger JN Mathew MS Marroquin EM Lingvay I. Effectiveness of semaglutide versus liraglutide for treating post-metabolic and bariatric surgery weight recurrence. Obesity (Silver Spring, Md). (2023) 31:1280–9. doi: 10.1002/oby.23736, PMID: 36998152 PMC12398307

[57] Istfan NW Anderson WA Hess DT Yu L Carmine B Apovian CM. The mitigating effect of phentermine and Topiramate on weight regain after Roux-en-Y gastric bypass surgery. Obesity (Silver Spring, Md). (2020) 28:1023–30. doi: 10.1002/oby.22786, PMID: 32441476 PMC7250052

[58] Toth AT Gomez G Shukla AP Pratt JS Cena H Biino G. Weight loss medications in young adults after bariatric surgery for weight regain or inadequate weight loss: a multi-center study. Children (Basel, Switzerland). (2018) 5:116. doi: 10.3390/children5090116 30158481 PMC6162731

[59] Grilo CM Reas DL Mitchell JE. Combining pharmacological and psychological treatments for binge eating disorder: current status, limitations, and future directions. Curr Psychiatry Rep. (2016) 18:55. doi: 10.1007/s11920-016-0696-z, PMID: 27086316

[60] Stanford FC Alfaris N Gomez G Ricks ET Shukla AP Corey KE. The utility of weight loss medications after bariatric surgery for weight regain or inadequate weight loss: a multi-center study. Surg Obes Relat Dis. (2017) 13:491–500. doi: 10.1016/j.soard.2016.10.018, PMID: 27986587 PMC6114136

[61] Greenway FL Fujioka K Plodkowski RA Mudaliar S Guttadauria M Erickson J. Effect of naltrexone plus bupropion on weight loss in overweight and obese adults (COR-I): a multicentre, randomised, double-blind, placebo-controlled, phase 3 trial. Lancet (London, England). (2010) 376:595–605. doi: 10.1016/S0140-6736(10)60888-4, PMID: 20673995

[62] Avenell A Robertson C Skea Z Jacobsen E Boyers D Cooper D. Bariatric surgery, lifestyle interventions and orlistat for severe obesity: the REBALANCE mixed-methods systematic review and economic evaluation. Health Technol Assessment (Winchester, England). (2018) 22:1–246. doi: 10.3310/hta22680, PMID: 30511918 PMC6296173

[63] Abu Dayyeh BK Rajan E Gostout CJ. Endoscopic sleeve gastroplasty: a potential endoscopic alternative to surgical sleeve gastrectomy for treatment of obesity. Gastrointest Endosc. (2013) 78:530–5. doi: 10.1016/j.gie.2013.04.197, PMID: 23711556

[64] Sharaiha RZ Kedia P Kumta N Aronne LJ Kahaleh M. Endoscopic sleeve plication for revision of sleeve gastrectomy. Gastrointest Endosc. (2015) 81:1004. doi: 10.1016/j.gie.2014.06.008, PMID: 25016406

[65] Eid G. Sleeve gastrectomy revision by endoluminal sleeve plication gastroplasty: a small pilot case series. Surg Endosc. (2017) 31:4252–5. doi: 10.1007/s00464-017-5469-1, PMID: 28364152

[66] Jirapinyo P de Moura DTH Thompson CC. Sleeve in sleeve: endoscopic revision for weight regain after sleeve gastrectomy. VideoGIE. (2019) 4:454–7. doi: 10.1016/j.vgie.2019.07.003, PMID: 31709328 PMC6831909

[67] Jirapinyo P Dayyeh BK Thompson CC. Gastrojejunal anastomotic reduction for weight regain in roux-en-y gastric bypass patients: physiological, behavioral, and anatomical effects of endoscopic suturing and sclerotherapy. Surg Obes Relat Dis. (2016) 12:1810–6. doi: 10.1016/j.soard.2016.09.036, PMID: 27998543 PMC5178872

[68] Kumar N Thompson CC. Comparison of a superficial suturing device with a full-thickness suturing device for transoral outlet reduction (with videos). Gastrointest Endosc. (2014) 79:984–9. doi: 10.1016/j.gie.2014.02.006, PMID: 24721521 PMC5038592

[69] Thompson CC Chand B Chen YK DeMarco DC Miller L Schweitzer M. Endoscopic suturing for transoral outlet reduction increases weight loss after Roux-en-Y gastric bypass surgery. Gastroenterology. (2013) 145:129–37.e3. doi: 10.1053/j.gastro.2013.04.002, PMID: 23567348

[70] Schulman AR Kumar N Thompson CC. Transoral outlet reduction: a comparison of purse-string with interrupted stitch technique. Gastrointest Endosc. (2018) 87:1222–8. doi: 10.1016/j.gie.2017.10.034, PMID: 29108984 PMC5899924

[71] Bulajic M Vadalà di Prampero SF Boškoski I Costamagna G. Endoscopic therapy of weight regain after bariatric surgery. World J Gastrointest Surg. (2021) 13:1584–96. doi: 10.4240/wjgs.v13.i12.1584, PMID: 35070065 PMC8727177

[72] Thompson CC Slattery J Bundga ME Lautz DB. Peroral endoscopic reduction of dilated gastrojejunal anastomosis after Roux-en-Y gastric bypass: a possible new option for patients with weight regain. Surg Endosc. (2006) 20:1744–8. doi: 10.1007/s00464-006-0045-0, PMID: 17024527

[73] Horgan S Jacobsen G Weiss GD Oldham JS Jr Denk PM Borao F. Incisionless revision of post-Roux-en-Y bypass stomal and pouch dilation: multicenter registry results. Surg Obes Relat Dis. (2010) 6:290–5. doi: 10.1016/j.soard.2009.12.011, PMID: 20510293

[74] Franken RJ Sluiter NR Franken J de Vries R Souverein D Gerdes VEA. Treatment options for weight regain or Insufcient weight loss after sleeve gastrectomy: a systematic review and Meta-analysis. Obes Surg. (2022) 32:2035–46. doi: 10.1007/s11695-022-06020-0, PMID: 35366738

[75] de Moura DTH de Moura EGH Thompson CC. Endoscopic sleeve gastroplasty: from whence we came and where we are going. World J Gastrointest Endoscopy. (2019) 11:322–8. doi: 10.4253/wjge.v11.i5.322, PMID: 31205593 PMC6556490

[76] Sharaiha RZ Kumta NA Saumoy M Desai AP Sarkisian AM Benevenuto A. Endoscopic sleeve Gastroplasty significantly reduces body mass index and metabolic complications in obese patients. Clin Gastroenterol Hepatol. (2017) 15:504–10. doi: 10.1016/j.cgh.2016.12.012, PMID: 28017845

[77] Langer FB Bohdjalian A Felberbauer FX Fleischmann E Reza Hoda MA Ludvik B. Does gastric dilatation limit the success of sleeve gastrectomy as a sole operation for morbid obesity? Obes Surg. (2006) 16:166–71. doi: 10.1381/096089206775565276, PMID: 16469218

[78] El Khoury L Catheline JM Taher M Roussel J Bendacha Y Romero R. Re-sleeve gastrectomy is a safe and sensible intervention in selected patients: retrospective cohort study. Int J Surg (London, England). (2023) 109:4145–50. doi: 10.1097/JS9.0000000000000743, PMID: 37707529 PMC10720822

[79] Bonaldi M Rubicondo C Giorgi R Cesana G Ciccarese F Uccelli M. Re-sleeve gastrectomy: weight loss, comorbidities and gerd evaluation in a large series with 5 years of follow-up. Updat Surg. (2023) 75:959–65. doi: 10.1007/s13304-023-01471-1, PMID: 36849646

[80] Chakhtoura G Zinzindohoué F Ghanem Y Ruseykin I Dutranoy JC Chevallier JM. Primary results of laparoscopic mini-gastric bypass in a French obesity-surgery specialized university hospital. Obes Surg. (2008) 18:1130–3. doi: 10.1007/s11695-008-9594-8, PMID: 18566866

[81] Jia D Tan H Faramand A Fang F. One anastomosis gastric bypass versus Roux-en-Y gastric bypass for obesity: a systematic review and Meta-analysis of randomized clinical trials. Obes Surg. (2020) 30:1211–8. doi: 10.1007/s11695-019-04288-3, PMID: 31749109

[82] Robert M Espalieu P Pelascini E Caiazzo R Sterkers A Khamphommala L. Efficacy and safety of one anastomosis gastric bypass versus Roux-en-Y gastric bypass for obesity (YOMEGA): a multicentre, randomised, open-label, non-inferiority trial. Lancet (London, England). (2019) 393:1299–309. doi: 10.1016/S0140-6736(19)30475-1, PMID: 30851879

[83] Wysocki M Borys M Budzyńska D Pisarska-Adamczyk M Małczak P Rajtar A. Initial experience with laparoscopic revisional single anastomosis duodeno-ileal bypass (SADI-S) after failed sleeve gastrectomy. Wideochir Inne Tech Maloinwazyjne. (2023) 18:298–304. doi: 10.5114/wiitm.2023.128683, PMID: 37680742 PMC10481443

[84] Homan J Betzel B Aarts EO van Laarhoven KJ Janssen IM Berends FJ. Secondary surgery after sleeve gastrectomy: Roux-en-Y gastric bypass or biliopancreatic diversion with duodenal switch. Surg Obes Relat Dis. (2015) 11:771–7. doi: 10.1016/j.soard.2014.09.029, PMID: 25769402

[85] Casillas RA Um SS Zelada Getty JL Sachs S Kim BB. Revision of primary sleeve gastrectomy to Roux-en-Y gastric bypass: indications and outcomes from a high-volume center. Surg Obes Relat Dis. (2016) 12:1817–25. doi: 10.1016/j.soard.2016.09.038, PMID: 27887931

[86] Franken RJ Franken J Sluiter NR de Vries R Euser S Gerdes VEA. Efficacy and safety of revisional treatments for weight regain or insufficient weight loss after Roux-en-Y gastric bypass: a systematic review and meta-analysis. Obes Rev. (2023) 24:e13607. doi: 10.1111/obr.13607, PMID: 37515352

[87] Razzak FA Kerbage A Brunaldi VO Mrad R Mahmoud T Gala K. Correlation between Gastrojejunal anastomosis diameter, Distensibility index, and weight regain after Roux-en-Y gastric bypass. Obes Surg. (2023) 33:4042–8. doi: 10.1007/s11695-023-06918-3, PMID: 37922061

[88] Arica PC Aydin S Zengin U Kocael A Orhan A Zengin K. The effects on obesity related peptides of laparoscopic gastric band applications in morbidly obese patients. J Investigat Surg. (2018) 31:89–95. doi: 10.1080/08941939.2017.1280564, PMID: 28635510

[89] Ng PC Sharp LS Bermudez DM. Duodenal switch: fully stapled technique. Surg Obes Relat Dis. (2019) 15:512. doi: 10.1016/j.soard.2018.12.031, PMID: 30765292

[90] Shin RD Goldberg MB Shafran AS Shikora SA Majumdar MC Shikora SA. Revision of Roux-en-Y gastric bypass with limb Distalization for inadequate weight loss or weight regain. Obes Surg. (2019) 29:811–8. doi: 10.1007/s11695-018-03635-0, PMID: 30560312

[91] Liao EP. Management of type 2 diabetes: new and future developments in treatment. Am J Med. (2012) 125:S2–3. doi: 10.1016/j.amjmed.2012.05.008, PMID: 22998892

[92] Sjöström L. Review of the key results from the Swedish obese subjects (SOS) trial - a prospective controlled intervention study of bariatric surgery. J Intern Med. (2013) 273:219–34. doi: 10.1111/joim.12012, PMID: 23163728

[93] Schauer PR Bhatt DL Kirwan JP Wolski K Aminian A Brethauer SA. Bariatric surgery versus intensive medical therapy for Diabetes - 5-year outcomes. N Engl J Med. (2017) 376:641–51. doi: 10.1056/NEJMoa1600869, PMID: 28199805 PMC5451258

[94] Gardner CD Trepanowski JF Del Gobbo LC Hauser ME Rigdon J Ioannidis JPA. Effect of low-fat vs low-carbohydrate diet on 12-month weight loss in overweight adults and the Association with genotype pattern or insulin secretion: the DIETFITS randomized clinical trial. JAMA. (2018) 319:667–79. doi: 10.1001/jama.2018.0245, PMID: 29466592 PMC5839290

[95] Pharmacologic Approaches to Glycemic Treatment. Standards of Care in Diabetes-2024. Diabetes Care. (2024) 47:S158–78. doi: 10.2337/dc24-S009, PMID: 38078590 PMC10725810

[96] Verspohl EJ. Novel therapeutics for type 2 diabetes: incretin hormone mimetics (glucagon-like peptide-1 receptor agonists) and dipeptidyl peptidase-4 inhibitors. Pharmacol Ther. (2009) 124:113–38. doi: 10.1016/j.pharmthera.2009.06.002, PMID: 19545590

[97] Bailey CJ. Renal glucose reabsorption inhibitors to treat diabetes. Trends Pharmacol Sci. (2011) 32:63–71. doi: 10.1016/j.tips.2010.11.011, PMID: 21211857

[98] Miller RA Chu Q Xie J Foretz M Viollet B Birnbaum MJ. Biguanides suppress hepatic glucagon signalling by decreasing production of cyclic AMP. Nature. (2013) 494:256–60. doi: 10.1038/nature11808, PMID: 23292513 PMC3573218

[99] Mulherin AJ Oh AH Kim H Grieco A Lauffer LM Brubaker PL. Mechanisms underlying metformin-induced secretion of glucagon-like peptide-1 from the intestinal L cell. Endocrinology. (2011) 152:4610–9. doi: 10.1210/en.2011-1485, PMID: 21971158

[100] Tian YA Johnson G Ashcroft SJ. Sulfonylureas enhance exocytosis from pancreatic beta-cells by a mechanism that does not involve direct activation of protein kinase C. Diabetes. (1998) 47:1722–6. doi: 10.2337/diabetes.47.11.1722, PMID: 9792541

[101] Glycemic Goals and Hypoglycemia. Standards of Care in Diabetes-2024. Diabetes Care. (2024) 47:S111–s25. 38078586 10.2337/dc24-S006 PMC10725808

[102] Comprehensive Medical Evaluation and Assessment of Comorbidities. Standards of medical Care in Diabetes-2022. Diabetes Care. (2022) 45:S46–s59. 34964869 10.2337/dc22-S004 PMC8935396

[103] Cardiovascular Disease and Risk Management. Standards of Care in Diabetes-2024. Diabetes Care. (2024) 47:S179–s218. 38078592 10.2337/dc24-S010 PMC10725811

[104] ElSayed NA Aleppo G Aroda VR Bannuru RR Brown FM Bruemmer D. Chronic kidney disease and risk management: standards of Care in Diabetes-2023. Diabetes Care. (2023) 46:S191–s202. doi: 10.2337/dc23-S011, PMID: 36507634 PMC9810467

[105] Yan J Cohen R Aminian A. Reoperative bariatric surgery for treatment of type 2 diabetes mellitus. Surg Obes Relat Dis. (2017) 13:1412–21. doi: 10.1016/j.soard.2017.04.019, PMID: 28647410

[106] Aleassa EM Hassan M Hayes K Brethauer SA Schauer PR Aminian A. Effect of revisional bariatric surgery on type 2 diabetes mellitus. Surg Endosc. (2019) 33:2642–8. doi: 10.1007/s00464-018-6541-1, PMID: 30341657

[107] Yale CE. Conversion surgery for morbid obesity: complications and long-term weight control. Surgery. (1989) 106:474–80. PMID: 2772823

[108] Linner JH Drew RL. Reoperative surgery—indications, efficacy, and long-term follow-up. Am J Clin Nutr. (1992) 55:606s–10s. doi: 10.1093/ajcn/55.2.606s, PMID: 1733138

[109] Behrns KE Smith CD Kelly KA Sarr MG. Reoperative bariatric surgery. Lessons learned to improve patient selection and results. Ann Surg. (1993) 218:646–53. doi: 10.1097/00000658-199321850-00010, PMID: 8239779 PMC1243036

[110] Gagné DJ Dovec E Urbandt JE. Laparoscopic revision of vertical banded gastroplasty to Roux-en-Y gastric bypass: outcomes of 105 patients. Surg Obes Relat Dis. (2011) 7:493–9. doi: 10.1016/j.soard.2010.10.014, PMID: 21195675

[111] Sarhan MD AbdelSalam NM Mostafa MS Yehia A Anwar I Fathy E. Laparoscopic Roux-en-Y gastric bypass after failed vertical banded Gastroplasty: 2-year follow-up of 102 patients. Obes Surg. (2021) 31:2717–22. doi: 10.1007/s11695-021-05328-7, PMID: 33660155

[112] Ngiam KY Khoo VY Kong L Cheng AK. Laparoscopic adjustable gastric banding revisions in Singapore: a 10-year experience. Obes Surg. (2016) 26:1069–74. doi: 10.1007/s11695-015-1852-y, PMID: 26314350

[113] Vidal J Ibarzabal A Romero F Delgado S Momblán D Flores L. Type 2 diabetes mellitus and the metabolic syndrome following sleeve gastrectomy in severely obese subjects. Obes Surg. (2008) 18:1077–82. doi: 10.1007/s11695-008-9547-2, PMID: 18521701

[114] Yeung L Durkan B Barrett A Kraft C Vu K Phillips E. Single-stage revision from gastric band to gastric bypass or sleeve gastrectomy: 6- and 12-month outcomes. Surg Endosc. (2016) 30:2244–50. doi: 10.1007/s00464-015-4498-x, PMID: 26335074

[115] Lee WJ Almulaifi A Tsou JJ Ser KH Lee YC Chen SC. Laparoscopic sleeve gastrectomy for type 2 diabetes mellitus: predicting the success by ABCD score. Surg Obes Relat Dis. (2015) 11:991–6. doi: 10.1016/j.soard.2014.12.027, PMID: 25868836

[116] Rawlins ML Teel D 2nd Hedgcorth K Maguire JP. Revision of Roux-en-Y gastric bypass to distal bypass for failed weight loss. Surg Obes Relat Dis. (2011) 7:45–9. doi: 10.1016/j.soard.2010.08.013, PMID: 21111688

[117] Pi-Sunyer X Astrup A Fujioka K Greenway F Halpern A Krempf M. A randomized, controlled trial of 3.0 mg of Liraglutide in weight management. N Engl J Med. (2015) 373:11–22. doi: 10.1056/NEJMoa1411892, PMID: 26132939

[118] Wilding JPH Batterham RL Calanna S Davies M Van Gaal LF Lingvay I. Once-weekly Semaglutide in adults with overweight or obesity. N Engl J Med. (2021) 384:989–1002. doi: 10.1056/NEJMoa2032183, PMID: 33567185

[119] Aronne LJ Wadden TA Peterson C Winslow D Odeh S Gadde KM. Evaluation of phentermine and topiramate versus phentermine/topiramate extended-release in obese adults. Obesity (Silver Spring, Md). (2013) 21:2163–71. doi: 10.1002/oby.20584, PMID: 24136928

[120] Garvey WT Ryan DH Look M Gadde KM Allison DB Peterson CA. Two-year sustained weight loss and metabolic benefits with controlled-release phentermine/topiramate in obese and overweight adults (SEQUEL): a randomized, placebo-controlled, phase 3 extension study. Am J Clin Nutr. (2012) 95:297–308. doi: 10.3945/ajcn.111.024927, PMID: 22158731 PMC3260065

[121] Sjöström L Rissanen A Andersen T Boldrin M Golay A Koppeschaar HP. Randomised placebo-controlled trial of orlistat for weight loss and prevention of weight regain in obese patients. European multicentre orlistat study group. Lancet (London, England). (1998) 352:167–72. doi: 10.1016/S0140-6736(97)11509-4 9683204

[122] Martínez-González MA Montero P Ruiz-Canela M Toledo E Estruch R Gómez-Gracia E. Yearly attained adherence to Mediterranean diet and incidence of diabetes in a large randomized trial. Cardiovasc Diabetol. (2023) 22:262. doi: 10.1186/s12933-023-01994-2, PMID: 37775736 PMC10542699

[123] Craig WJ Mangels AR. Position of the American dietetic Association: vegetarian diets. J Am Diet Assoc. (2009) 109:1266–82. doi: 10.1016/j.jada.2009.05.027, PMID: 19562864

[124] Wheeler ML Dunbar SA Jaacks LM Karmally W Mayer-Davis EJ Wylie-Rosett J. Macronutrients, food groups, and eating patterns in the management of diabetes: a systematic review of the literature, 2010. Diabetes Care. (2012) 35:434–45. doi: 10.2337/dc11-2216, PMID: 22275443 PMC3263899

[125] Krauss RM Eckel RH Howard B Appel LJ Daniels SR Deckelbaum RJ. AHA dietary guidelines: revision 2000: a statement for healthcare professionals from the nutrition Committee of the American Heart Association. Stroke. (2000) 31:2751–66. doi: 10.1161/01.STR.31.11.2751, PMID: 11062305

[126] Harsha DW Lin PH Obarzanek E Karanja NM Moore TJ Caballero B. Dietary approaches to stop hypertension: a summary of study results. DASH collaborative research group. J Am Diet Assoc. (1999) 99:S35–9. doi: 10.1016/S0002-8223(99)00414-9, PMID: 10450292

[127] Tsapas A Avgerinos I Karagiannis T Malandris K Manolopoulos A Andreadis P. Comparative effectiveness of glucose-lowering drugs for type 2 Diabetes: a systematic review and network Meta-analysis. Ann Intern Med. (2020) 173:278–86. doi: 10.7326/M20-0864, PMID: 32598218

[128] Tsapas A Karagiannis T Kakotrichi P Avgerinos I Mantsiou C Tousinas G. Comparative efficacy of glucose-lowering medications on body weight and blood pressure in patients with type 2 diabetes: a systematic review and network meta-analysis. Diabetes Obes Metab. (2021) 23:2116–24. doi: 10.1111/dom.14451, PMID: 34047443

[129] Botros FT Gerstein HC Malik R Nicolay C Hoover A Turfanda I. Dulaglutide and kidney function-related outcomes in type 2 Diabetes: a REWIND post hoc analysis. Diabetes Care. (2023) 46:1524–30. doi: 10.2337/dc23-0231, PMID: 37343574

[130] Herrington WG Haynes R. Diabetic kidney disease - Semaglutide flows into the mainstream. N Engl J Med. (2024) 391:178–9. doi: 10.1056/NEJMe2406408, PMID: 38986062

[131] Perkovic V Jardine MJ Neal B Bompoint S Heerspink HJL Charytan DM. Canagliflozin and renal outcomes in type 2 Diabetes and nephropathy. N Engl J Med. (2019) 380:2295–306. doi: 10.1056/NEJMoa1811744, PMID: 30990260

[132] Lu Q Yang L Xiao JJ Liu Q Ni L Hu JW. Empagliflozin attenuates the renal tubular ferroptosis in diabetic kidney disease through AMPK/NRF2 pathway. Free Radic Biol Med. (2023) 195:89–102. doi: 10.1016/j.freeradbiomed.2022.12.088, PMID: 36581059

[133] Wang YH Chang DY Zhao MH Chen M. Dapagliflozin alleviates diabetic kidney disease via hypoxia inducible factor 1α/Heme oxygenase 1-mediated Ferroptosis. Antioxid Redox Signal. (2024) 40:492–509. doi: 10.1089/ars.2022.0169, PMID: 37276148

[134] Verma S David JP Leiter LA Michelsen MM Rasmussen S Bhatt DL. Semaglutide reduces the risk of major adverse cardiovascular events consistently across baseline triglyceride levels in patients with type 2 diabetes: post hoc analyses of the SUSTAIN 6 and PIONEER 6 trials. Diabetes Obes Metab. (2023) 25:2388–92. doi: 10.1111/dom.15081, PMID: 37016488

[135] Verma S Bain SC Buse JB Idorn T Rasmussen S Ørsted DD. Occurence of first and recurrent major adverse cardiovascular events with Liraglutide treatment among patients with type 2 Diabetes and high risk of cardiovascular events: a post hoc analysis of a randomized clinical trial. JAMA Cardiol. (2019) 4:1214–20. doi: 10.1001/jamacardio.2019.3080, PMID: 31721979 PMC6865601

[136] Stenberg E Näslund E. Major adverse cardiovascular events among patients with type-2 diabetes, a nationwide cohort study comparing primary metabolic and bariatric surgery to GLP-1 receptor agonist treatment. Int J Obes. (2023) 47:251–6. doi: 10.1038/s41366-023-01254-z, PMID: 36670155 PMC10113141

[137] Weir MR Gogate J Damaraju CV Correa-Rotter R Mahaffey KW. Effects of canagliflozin on major adverse cardiovascular events by baseline estimated glomerular filtration rate: pooled Hispanic subgroup analyses from the CANVAS program and CREDENCE trial. Diabetes Obes Metab. (2022) 24:12–20. doi: 10.1111/dom.14539, PMID: 34463423

[138] Htoo PT Tesfaye H Schneeweiss S Wexler DJ Everett BM Glynn RJ. Cardiorenal effectiveness of empagliflozin vs. glucagon-like peptide-1 receptor agonists: final-year results from the EMPRISE study. Cardiovasc Diabetol. (2024) 23:57. doi: 10.1186/s12933-024-02150-0, PMID: 38331813 PMC10854040

[139] Roumie CL Chipman J Min JY Hackstadt AJ Hung AM Greevy RA Jr. Association of Treatment with Metformin vs sulfonylurea with major adverse cardiovascular events among patients with Diabetes and reduced kidney function. JAMA. (2019) 322:1167–77. doi: 10.1001/jama.2019.13206, PMID: 31536102 PMC6753652

[140] Wilcox R Kupfer S Erdmann E. Effects of pioglitazone on major adverse cardiovascular events in high-risk patients with type 2 diabetes: results from PROspective pioglitAzone clinical trial in macro vascular events (PROactive 10). Am Heart J. (2008) 155:712–7. doi: 10.1016/j.ahj.2007.11.029, PMID: 18371481

[141] Rådholm K Figtree G Perkovic V Solomon SD Mahaffey KW de Zeeuw D. Canagliflozin and heart failure in type 2 Diabetes mellitus: results from the CANVAS program. Circulation. (2018) 138:458–68. doi: 10.1161/CIRCULATIONAHA.118.034222, PMID: 29526832 PMC6075881

[142] Anker SD Butler J Filippatos G Ferreira JP Bocchi E Böhm M. Empagliflozin in heart failure with a preserved ejection fraction. N Engl J Med. (2021) 385:1451–61. doi: 10.1056/NEJMoa2107038, PMID: 34449189

[143] McMurray JJV Solomon SD Inzucchi SE Køber L Kosiborod MN Martinez FA. Dapagliflozin in patients with heart failure and reduced ejection fraction. N Engl J Med. (2019) 381:1995–2008. doi: 10.1056/NEJMoa1911303, PMID: 31535829

[144] Cannon CP Pratley R Dagogo-Jack S Mancuso J Huyck S Masiukiewicz U. Cardiovascular outcomes with Ertugliflozin in type 2 Diabetes. N Engl J Med. (2020) 383:1425–35. doi: 10.1056/NEJMoa2004967, PMID: 32966714

[145] Scirica BM Bhatt DL Braunwald E Steg PG Davidson J Hirshberg B. Saxagliptin and cardiovascular outcomes in patients with type 2 diabetes mellitus. N Engl J Med. (2013) 369:1317–26. doi: 10.1056/NEJMoa1307684, PMID: 23992601

[146] Singh S Loke YK Furberg CD. Thiazolidinediones and heart failure: a teleo-analysis. Diabetes Care. (2007) 30:2148–53. doi: 10.2337/dc07-0141, PMID: 17536074

